# Electrowetting-on-dielectric characteristics of ZnO nanorods

**DOI:** 10.1038/s41598-020-71017-7

**Published:** 2020-08-25

**Authors:** Jae-Hun Kim, Jae-Hyoung Lee, Ali Mirzaei, Hyoun Woo Kim, Boon Teoh Tan, Ping Wu, Sang Sub Kim

**Affiliations:** 1 Department of Materials Science and Engineering, Inha University, Incheon, 22212 Republic of Korea; 2 Department of Materials Science and Engineering, Shiraz University of Technology, 71557-13876 Shiraz, Iran; 3 The Research Institute of Industrial Science, Hanyang University, Seoul, 04763 Republic of Korea; 4 Division of Materials Science and Engineering, Hanyang University, Seoul, 04763 Republic of Korea; 5 Entropic Interface Group, Singapore University of Technology and Design, Singapore, 487372 Singapore

**Keywords:** Materials science, Nanoscience and technology

## Abstract

Herein, we report the electrowetting-on-dielectric (EWOD) characteristics of ZnO nanorods (NRs) prepared via the hydrothermal method with different initial Zn^2+^ concentrations (0.03, 0.07, and 0.1 M). Diameter of the resultant ZnO NRs were 50, 70 and 85 nm, respectively. Contact angle (CA) measurements showed that the Teflon-coated ZnO NRs with diameters of 85 nm prepared from the 0.1 M solution had the highest CA (137°). During the EWOD studies, on the application of a voltage of 250 V, the water CA decreased to 78°, which demonstrates the potential application of this material in EWOD electronics. Furthermore, we explained the relationship between the applied voltage and CA based on the substrate nanostructures and our newly developed NR-on-film wetting model. In addition, we further validated our model by introducing the homo-composite dielectric structure, which is a composite of thin layered ZnO/Teflon and nano-roded ZnO/Teflon.

## Introduction

**Figure 1:**
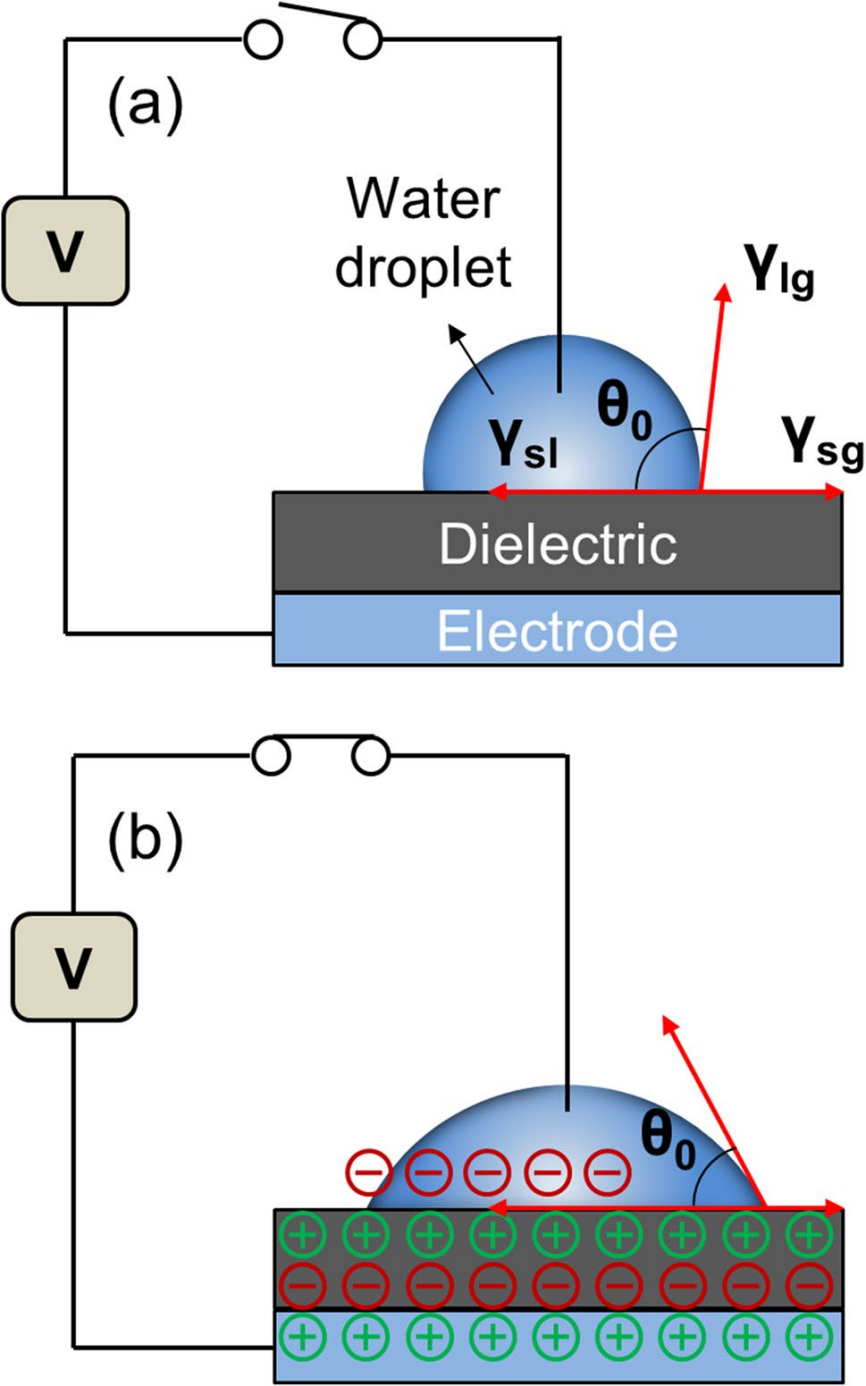
Principle of EWOD. (**a**) Without applied voltage, the surface shows hydrophobic state where wetting angle (θ_0_) is larger than 90°. (**b**) With an applied voltage, EWOD can be induced by decrease of wetting angle. γ_lg_, γ_sg_, and γ_sl_ are the liquid–air, solid–air, and solid–liquid interfacial energies, respectively.

Electrowetting (EW) is defined as the decrease in contact angle (CA) when a sufficiently large driving voltage is applied to the interface of solid/liquid. In direct EW, a voltage is used between a liquid and an electrode. Charges and dipoles redistribute at the interface between the liquid and solid, which changes the surface tension, leading to a decrease in the CA of the liquid droplets. However, direct EW can electrolyze the liquid before any change in the water contact angle (WCA), which limits its application^1^. To overcome this issue, electrowetting-on-dielectric (EWOD) can be used. In this technique, a dielectric material is sandwiched between the liquid of interest and the underlying electrode (Fig. 1). EWOD is very effective for significantly changing the CA, because the dielectric layer effectively blocks the process of charge transfer at the liquid/electrode interface, which eliminates unwanted electrolysis^2^. Therefore, EWOD is used to facilitate EW on solids, for practical applications.

Although EWOD devices permit a large CA change, they generally need a high driving voltage. The dielectric layer blocks the transfer of electrons, while sustaining high voltage at the interface, which results in an electric double layer (EDL) formation in the presence of the applied voltage. When a hydrophobic dielectric material is used, the large initial WCA can result in a large change in the WCA^3^. The advantages of EWOD include low power consumption, large WCA change, and fast response. It is therefore promising for diverse applications, including lab-on-chips^4^, liquid lenses^5^, optical waveguides^6^, electronic displays^7, 8^, microprism arrays^9^, and smart microbatteries^10^.

SiO_2_ is a highly popular material for EWOD studies^11^ because of its excellent compatibility with the microelectronic industry. In our previous study^12^, we investigated the EWOD properties of electrospray-deposited SiO_2_ layers and found that the WCA of the optimal SiO_2_ layer underwent a hydrophobic-to-hydrophilic transition under a driving voltage of 150 V in air. A novel EWOD mechanism, wherein the induced moderate leakage current was considered, was also presented. Metal oxides are another popular material for EWOD studies, and their wettability is of significant practical interest^13, 14^. Unfortunately, there are few reports discussing EWOD on ZnO, which is a well-known metal oxide. ZnO has fascinating properties, such as piezoelectricity^15^, semiconductivity^16^, dielectricity^17^, and stable thermal/chemical and physical properties^18^. Accordingly, it is interesting to explore the EWOD properties of ZnO. Wu et al. reported on the EWOD properties of ZnO nanowires. Reversible EW was observed at low voltages (0–50 V), and an irreversible behavior was observed at higher voltages (50–100 V). Moreover, when applied voltage was increased from 0 to 100 V, the WCA was continuously decreased from 165° to 20° and exhibited instant reversibility^19^. Chen et al. investigated the EWOD properties of ZnO with bowl-like morphology, where the water droplet on semi-layered ZnO nanostructures underwent a transition from the Cassie–Baxter to the Wenzel state, while the Cassie–Baxter state, mixed Cassie–Baxter/Wenzel state, and Wenzel state were all found on multi-layered ZnO nanostructures^20^. Zhou et al. studied the EWOD properties of ZnO thin films prepared via electrochemical deposition and the effect of deposition condition on the EWOD properties^21^. Furthermore, a barrier layer with a high dielectric constant was combined with ZnO nanorods (NRs) (with thickness of 800 nm) to reach a WCA of 164.8° with a contact angle hysteresis of 11.3°. Sirbu et al. sputter-deposited ZnO NRs (400 nm) on glass for their EWOD studies^22^. Campbell et al. investigated the EWOD properties of ZnO NRs using different liquid-phase deposition techniques^23^. For high applied voltages, large CA changes were observed. Nevertheless, the surface was not reversible after the applied voltage was removed and required mechanical agitation to return to its initial state. Xia et al. prepared ZnO tetrapods with initial WCA of 155°, which was decreased upon application of external voltage^24^. Electrolysis was also avoided by applying a dielectric layer between the conductive substrate and the ZnO tetrapods.

Supplementary Table S1 summarizes some EWOD studies on different materials. Inspired by the few number of EWOD studies on one-dimensional ZnO morphologies, we report on the EWOD properties of Teflon-coated ZnO NRs obtained via the hydrothermal route.

It is generally accepted that 1D nanostructures are useful materials for investigating the dependence of electrical properties on dimensionality and size reduction (or quantum confinement)^25^. In fact, when a bulk semiconductor is reduced to an NW and NR, its electronic structures will be confined in two dimensions which will result in the increase of its bandgap and maybe the change in electronic structure type. For instance, bulk Si is an indirect semiconductor but an NW Si can possess a direct bandgap if its diameter is carefully reduced^26^. Among the 1D nanostructures, ZnO NRs and NWs have been widely studied because of their easy nanomaterials formation and device applications^27^. Both ZnO NR and ZnO NW are direct semiconductors, a major difference between NR and NW is the size of the cross-section; the smaller the size, the larger the bandgap. Therefore, to explore the nano-confinement effects to EWOD, we systematically investigated systems with 1-D confined nano-film^12^, 2-D confined NR (in current study), and 3-D confined quantum dots in the next publication. In particular, we expect the bandgap enlargement in the NR is smaller than that of the NW. According to our design, since the leaking current plays a critical role in the EWOD performance, we would prefer a controllable and adequate leaking current generated by the applied voltage. The expected leaking current when using ZnO NR is higher and maybe better controllable than that of ZnO NW, because of their bandgap difference. The exact physics is not clear at the moment which can be explored using our previous modeling approach^26^, by varying the diameter of the ZnO NR-NW models. Because we focus on EWOD experiments in this work, we did not discuss this size-dependent quantum confinement in this manuscript. Our plan is to revisit the physics after we published the 3D confined structures (quantum dot) in the next step. In summary, we applied NR to explore the 2D quantum confinement effect to EWOD, since NR is expected to have reduced electronic bandgap and higher leaking current than these of ZnO NW.

Teflon-coated ZnO NRs which were hydrothermally grown on a ZnO layer were used for realization of EWOD platform. Effect of diameter of ZnO NRs on the surface roughness and WCA was investigated. It was found that the WCA decreases with increasing external voltage, which is explained based on the substrate nanostructures and the newly developed NR-on-film wetting model. Our work advances the fundamental understanding of the EWOD principle and generates new practical techniques for reversible WCA changes in a large WCA range. We revealed two ionization/polarization process respectively sourced from ZnO NRs and ZnO NRs along with ZnO thin films. Our work may inspire new techniques for nondestructive characterization of nanomaterials, nanostructures, and nano-processing.

## Results

### Morphological studies

**Figure 2:**
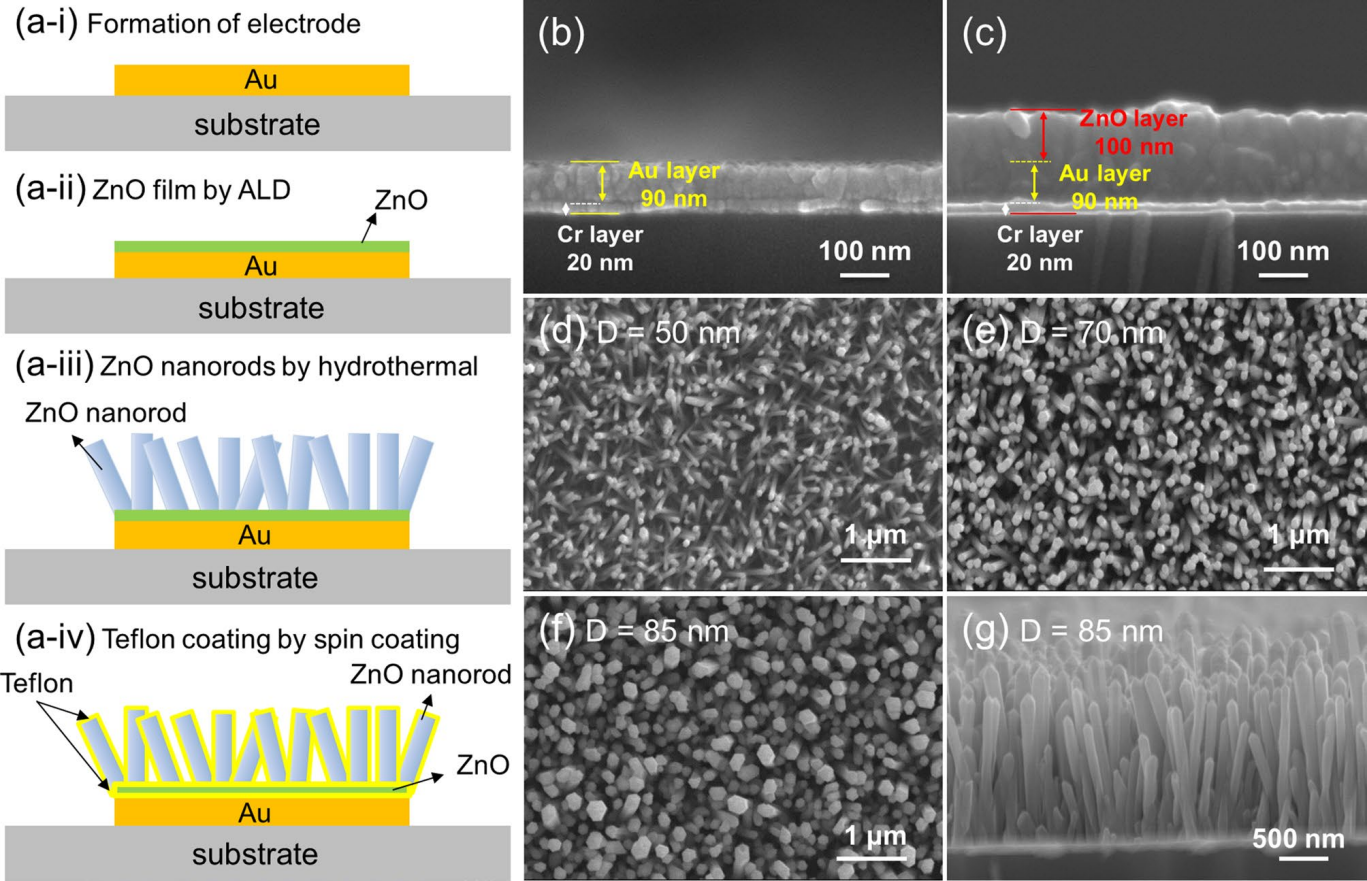
(**a-i**)–(**a-iv**) Schematic illustration of synthesis steps of Teflon-coated ZnO NRs. FE-SEM micrographs of (**b**) electroded substrate and (**c**) ZnO deposited layer on the substrate. Top-view FE-SEM micrographs of ZnO NRs with different diameters of (**d**) 50 nm (**e**) 70 nm and (**f**) 85 nm. (**g**) Cross-sectional FE-SEM image of ZnO NRs with diameter of 85 nm, prepared from Zn^2+^ solution with initial concentration of 0.1 M.

Figure 2a-i–a-iv schematically show the processing steps for the preparation of Teflon-coated ZnO NRs. In the following, each step is described in detail. Figure 2b shows cross-sectional FE-SEM images of electroded substrate, demonstrating deposition of Cr and Au layers with thicknesses of 20 and 90 nm, respectively. On the other hand, Fig. 2c reveals a deposited ZnO layer with a thickness of 100 nm on the substrate.

Supplementary Fig. S1 shows a representative XRD pattern obtained from a ZnO seed layer grown by ALD. It shows the peaks corresponding to (100), (002), (101) and (102) planes of hexagonal wurtzite ZnO (JCDPS Card # 89-1397)^28^. The wurtzite structure of ZnO contains polar and nonpolar facets with different surface energies, which is responsible for the faster growth rate of one crystallographic facet over another, namely anisotropic growth^29^. The relatively stronger intensity of the (002) peak demonstrates a preferential growth in that direction in some degree. The preferential growth is due to the fact that the c-plane which is perpendicular to the substrate is the most densely packed and thermodynamically favorable plane in the wurtzite structure^30^. In addition, the broad XRD peaks shown in Supplementary Fig. S1 indicate the nanocrystalline nature of the ZnO seed layer^31^.

Supplementary Fig. S2 shows cross-sectional FE-SEM images of ZnO NRs prepared from different initial concentrations of 0.03, 0.07, and 0.1 M. As shown, the diameter of ZnO NRs prepared with above concentrations are 50, 70 and 85 nm, respectively. Therefore, by changing of initial Zn^2+^ concentration it was possible to control the final diameters of the synthesized ZnO NRs. Figure 2d–f display top-view FE-SEM micrographs, with different magnifications, of ZnO NRs with different diameters of 50, 70 and 85 nm, prepared from the 0.03, 0.07, and 0.1 M initial Zn^2+^ solutions, respectively. All of them show dense NR morphologies with hexagonal cross sections. However, the packing densities of the NRs gradually increase with the increase in solution concentration, and the ZnO NRs with diameters of 85 nm, prepared from the 0.1 M solution demonstrate a more densely packed NR morphology. Furthermore, the orientations of the ZnO NRs change from a randomly oriented morphology to a vertically aligned morphology with the increase in Zn^2+^ initial concentration. Figure 2g shows a cross-sectional FE-SEM micrograph of ZnO NRs with diameters of 85 nm, prepared from the Zn^2+^ solution with initial concentration of 0.1 M, demonstrating the success of the synthesis of ZnO NRs.

Growth of vertically-aligned ZnO NRs on a ZnO seed layer by the hydrothermal method has been reported previously^32–35^. ZnO NRs with c-axis preferential orientation usually grow on lattice-matched ZnO (0001) layer by following the layer-by-layer growth mode^33^. It is also reported that the c-axis alignment of ZnO NRs is strongly dependent on the degree of preferential orientation of a ZnO seed layer^35^.

**Figure 3:**
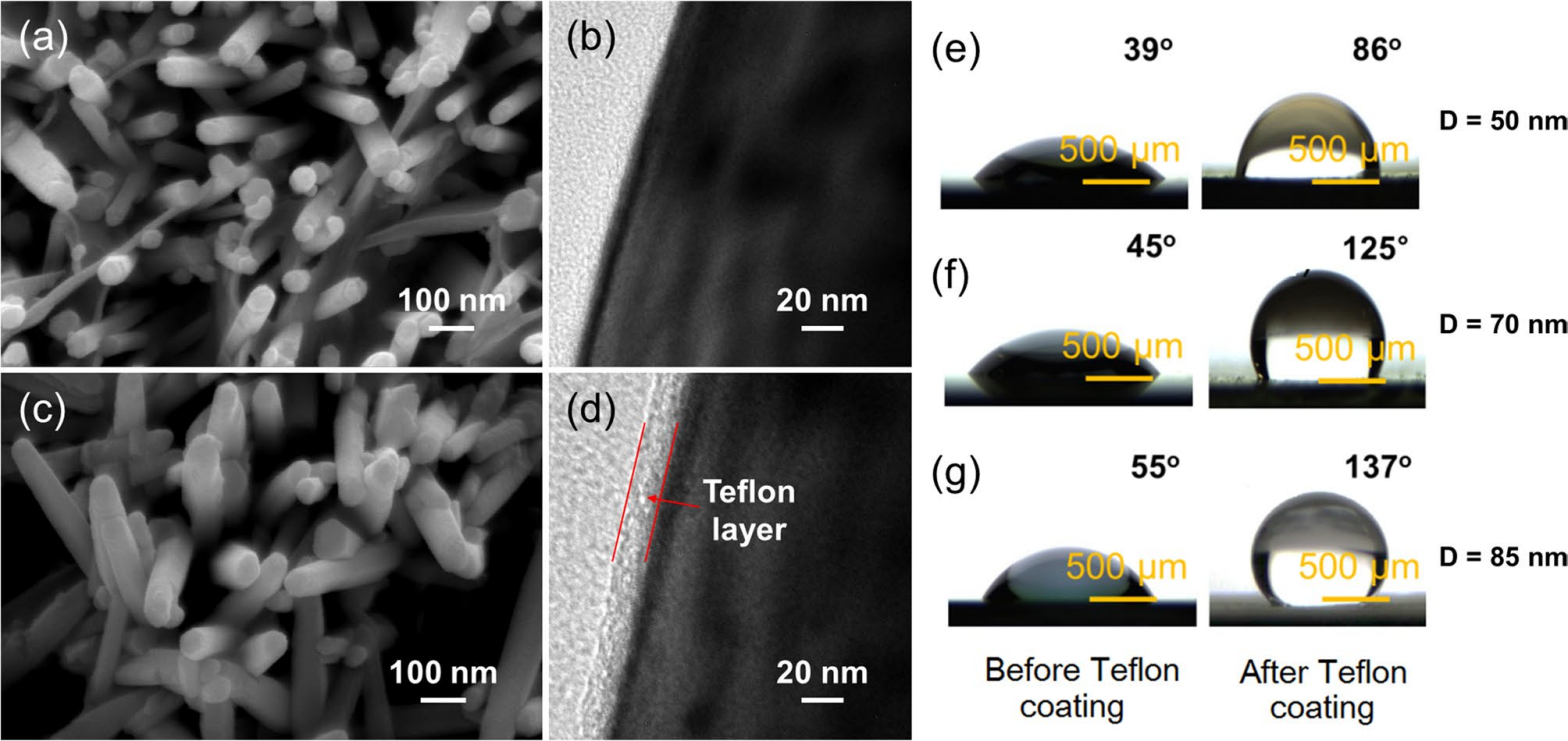
(**a**) FE-SEM and (**b**) TEM micrographs of ZnO NRs with diameters of 85 nm before Teflon coating. (**c**) TEM micrographs of ZnO NRs prepared with diameters of 85 nm after Teflon coating. WCAs for the samples before and after Teflon coating for ZnO NRs with different diameters of (**d**) 50 nm (**e**) 70 nm, and (**f**) 85 nm. Magnification × 5.

Figure 3a,c display FE-SEM images of ZnO NRs with diameters of 85 nm, before and after Teflon coating, respectively. To further study the thickness of the Teflon layer, TEM micrographs were obtained. Figure 3b shows a TEM image of ZnO NRs before Teflon coating, while Fig. 3d shows a TEM image after Teflon coating. The thickness of the Teflon layer, according to the TEM image, is about 20 nm. A number of ZnO NRs were randomly selected and observed to check the thickness uniformity of the Teflon layer by using TEM. As shown in Supplementary Fig. S3a–d, the Teflon layers showed a uniform thickness of ~ 20 nm (± 2 nm) in all cases. Therefore, it seems reasonable to conclude that there was overall uniformity in the Teflon coating, as shown in the TEM images.

In EWOD studies, the dielectric film prevents electrolysis of the water droplets. In addition, the dielectric film should have some special characteristics for smooth transition of the water droplet under applying of an external voltage. First, in order to induce larger change in WCA on actuation it should be hydrophobic, with a WCA > 90°. Second, to reduce applied voltage a film with higher dielectric constant is essential. But, most of the hydrophobic materials have low dielectric constant. So, generally a thin film of low energy hydrophobic layer such as Teflon is coated on top of the dielectric film to increase the initial WCA for EWOD studies and also to reduce applied voltage used for actuating of water droplets^12, 36, 37^.

**Figure 4:**
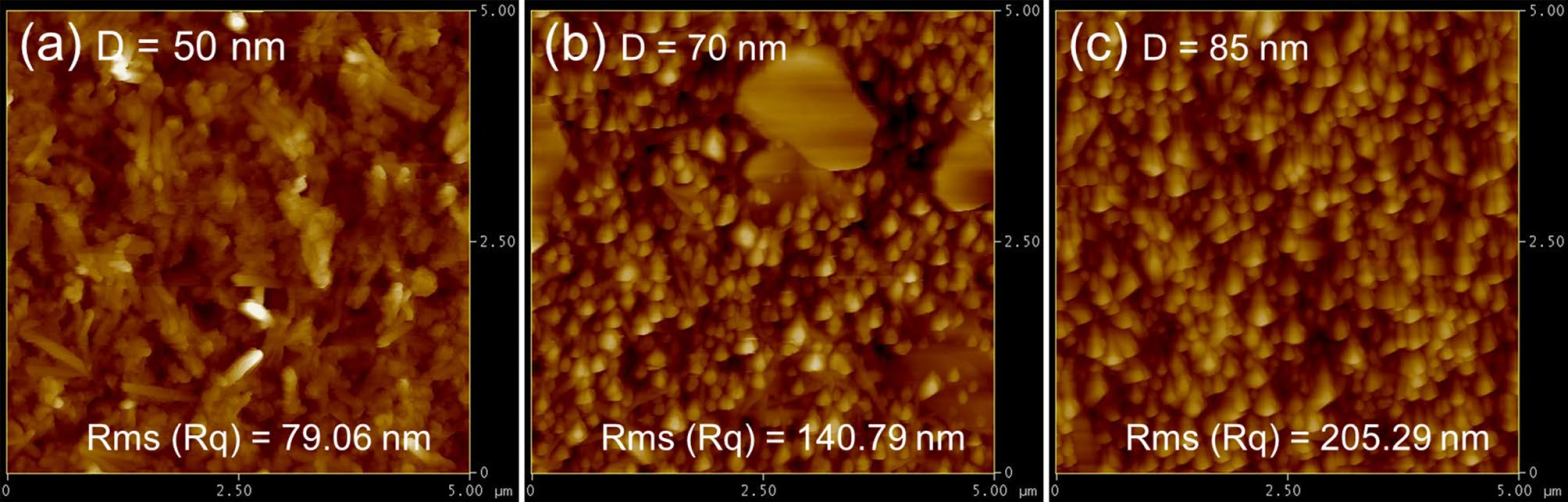
AFM images of ZnO NRs with different diameters of (**a**) 50 nm, (**b**) 70 nm and (**c**) 85 nm. Corresponding RMS values are 79.06, 140.79, and 205.29 nm, respectively.

The surface roughness values for different ZnO NRs are shown in Fig. 4. Based on the AFM images, the root-mean-squared (RMS) values for the ZnO NRs with different diameters of 50, 70 and 85 nm are 79.06, 140.79 and 205.29 nm, respectively. Because a higher surface roughness is more favorable for having superhydrophobic properties in case the wetting angle is greater than 90°, it is expected that the Teflon-coated ZnO NRs with diameters of 85 nm would demonstrate better superhydrophobic properties and better EWOD properties.

### Electrical behavior

A Schottky contact is usually made when the work function of a metal is lower (relative to vacuum) than that of an n-type semiconductor. ZnO is an n-type metal oxide with majority of carriers being electrons. The carrier concentration in ZnO depends on different parameters such as the morphology and growth method and accordingly, different values of carrier concentration are reported in the literature as shown in Supplementary Table S2. According to this table, overall, it can be concluded that for the ZnO NRs synthesized by the hydrothermal method used in this study, the carrier concentration has a fairly wide range. As a typical n-type metal oxide semiconductor, ZnO has a higher work function (4.2 eV) relative to Au (5.35 eV), possibly leading to the formation of a Schottky contact^38^. However, a weak Schottky contact behavior was observed probably due to the high doping level of ZnO NRs.

### EWOD studies

Because a larger initial CA is more favorable for EWOD studies, the WCAs on the surfaces of the prepared ZnO NRs were measured first. Figure 3e–g displays optical images of water droplets on the surfaces of prepared samples, before and after Teflon coating. In all cases, the WCA is seen to increase after coating of Teflon. For example, the WCAs before Teflon coating for the ZnO NRs with diameters of 50, 70 and 85 nm, are 39°, 45°, and 55°, respectively, which are greatly increased to 86°, 125°, and 137°, respectively, upon Teflon coating. This result demonstrates the effectiveness of Teflon coating on the wetting behavior of ZnO NRs. The ZnO NRs with diameters of 50 nm demonstrate hydrophilic properties, whereas the two other samples reveal a hydrophobic state. A possible reason for the lower WCAs for the samples with lower diameters is low surface roughness, as demonstrated in the AFM images (Fig. 4). It is well known that the larger the initial WCA, the greater the possible change in the WCA^23^. Accordingly, Teflon-coated ZnO NRs with diameters of 85 nm were chosen for the EWOD studies.

**Figure 5:**
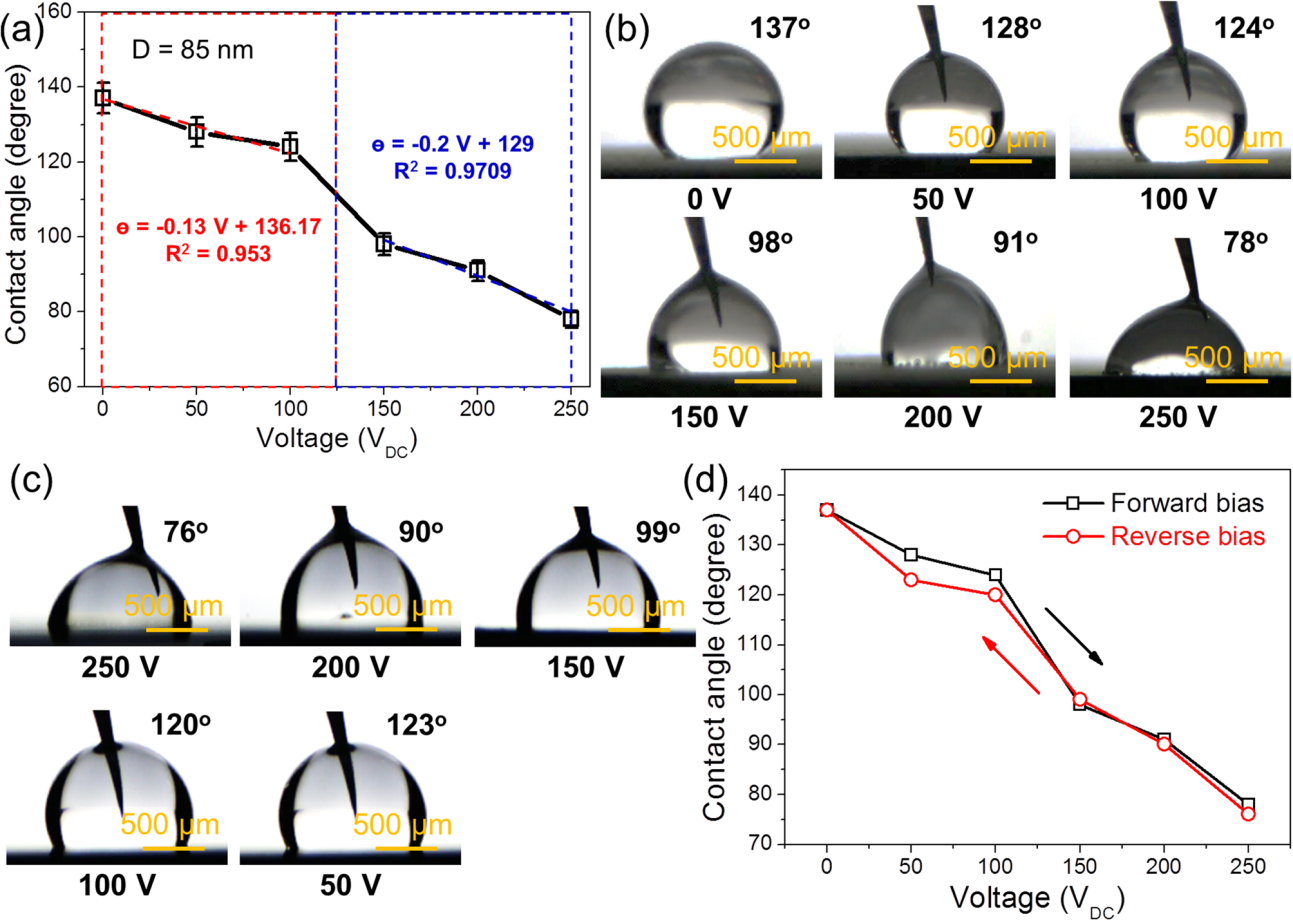
(**a**) Relationship between the WCAs and applied voltage for Teflon-coated ZnO NRs with diameters of 85 nm. (**b**) Optical images of water droplets on Teflon-coated ZnO NRs with diameters of 85 nm, under different external voltages and (**c**) during reverse bias voltages. Magnification × 5. (**d**) Experimental results of WCA hysteresis.

Figure 5a presents the variation in the WCAs as a function of applied voltage in the range of 0–250 V. The use of a direct linear relationship between WCA and voltage is guided by our new NR-on-film wetting model, which was reported in our previous work^12^. We established a linear relationship between WCA and system entropy and demonstrated that the system entropy associated with electrical leaking currents is a linear function of the voltage, not of the square of the voltage. Figure 5b displays optical images of the droplets under the different applied voltages. Supplementary Fig. S4 likewise shows optical images of the droplets under various applied voltages for the Teflon-coated ZnO NRs with diameters of 50 and 70 nm. For these samples, the initial WCA values are not very high because of lower surface roughness, so applied voltage did not significantly change the final WCA values. Thus, we focus on the result obtained for the Teflon-coated ZnO NRs with diameters of 85 nm. The DC voltage limit of the instrument used in the present study was 250 V, so we were not able to apply higher voltages. Apparently, the WCA was reduced continuously with increasing applied voltage. The initial WCA was 137°, which was decreased to 128°, 124°, 98°, 91°, and 78° under driving voltages of 50, 100, 150, 200, and 250 V, respectively. In other words, upon application of 250 V external voltage, the WCA was decreased by 59°. These results obey the well-known Lippmann–Young equation, which presents the relationship between the applied voltage (V) and WCA variations as follows^39–41^:

1 $$Cos\theta_{v} = cos\theta_{0} + \frac{1}{2}\frac{C}{{\gamma_{LG} }}V^{2} = cos\theta_{0} + \frac{1}{2}\frac{{\varepsilon \varepsilon_{0} }}{{\gamma_{LG} d}}V^{2} ,$$

where $\theta_{v}$ is the WCA in the presence of V, $\theta_{0}$ is the initial WCA, *c* is the specific capacitance, $\varepsilon_{0}$ and $\varepsilon$ are the dielectric permittivity of vacuum and the relative dielectric permittivity, respectively, *d* is the thickness of the dielectric material, and $\gamma_{LG}$ is the liquid–air interfacial tension. Equation (1) implies that when a voltage is applied, the WCA decreases, which is in accordance with our findings, although it fails to take into account the substrate nanostructures and leakage currents, which will be addressed in this study.

**Figure 6:**
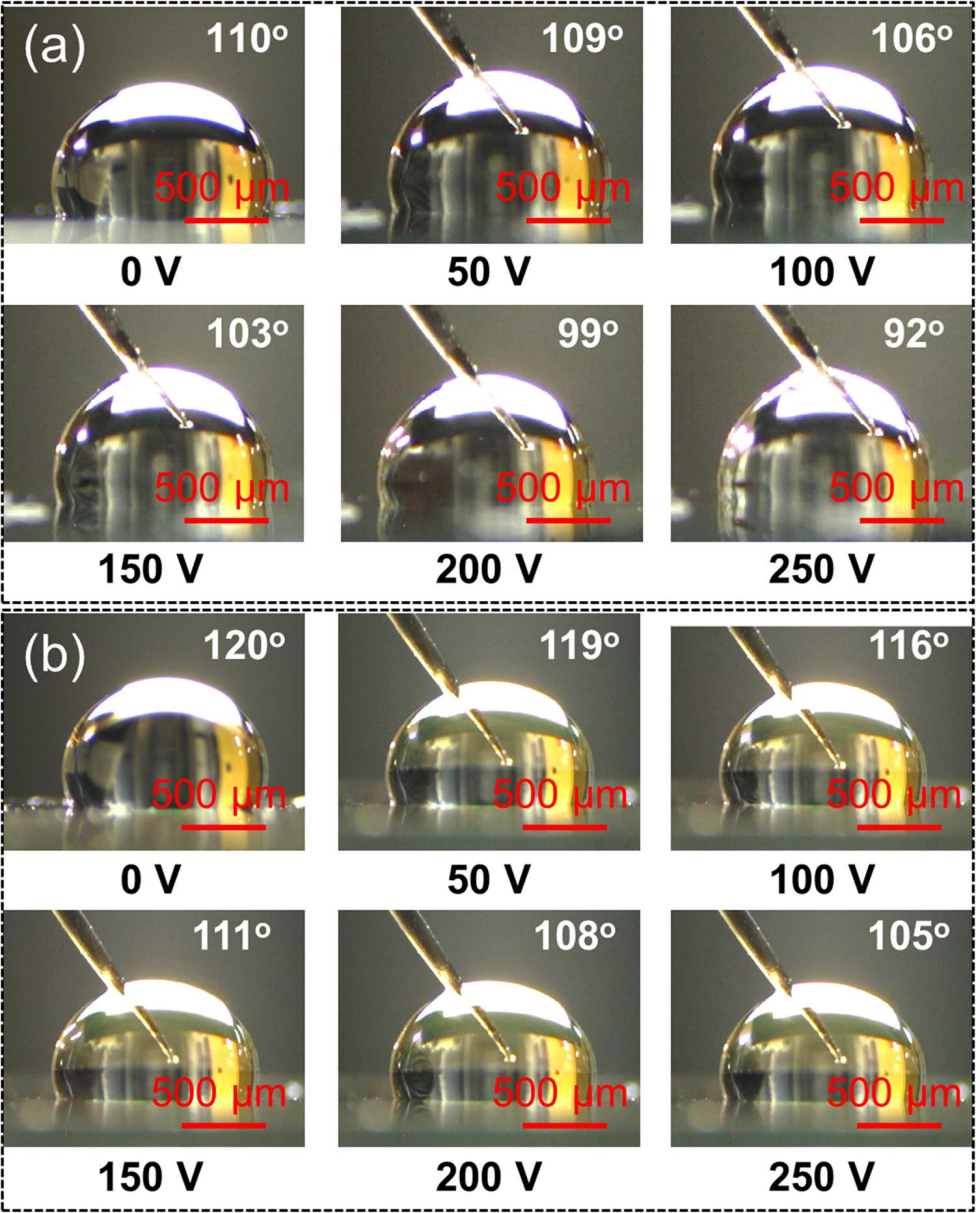
Optical images of water droplets on (**a**) Teflon-coated Au electrode (90 nm thick) and (**b**) Teflon-coated ZnO thin film (100 nm thick)/Au electrode (90 nm thick) under different external voltages.

For comparison, we also performed EW measurement for the samples: (1) Teflon-coated Au electrode and (2) Teflon-coated ZnO thin film/Au electrode. Figure 6a shows the optical images of water droplets on Teflon-coated Au electrode (90 nm thick) sample. Initially, with no applied voltage, the WCA is 110°, showing a hydrophobic nature. Upon applying voltages of 50, 100, 150, 200, and 250 V, the WCAs are gradually decreased to 109°, 106°, 103°, 99°, and 92°, respectively. Figure 6b shows optical images of water droplets on Teflon-coated ZnO thin film (100 nm thick)/Au electrode (90 nm thick) under different external voltages. The WCAs under external voltages of 0, 50, 100, 150, 200, and 250 V are 120°, 119°, 116°, 111°, 108°, and 105°, respectively. Therefore according to the above results, it can be concluded that both Teflon-coated Au electrode and Teflon-coated ZnO thin film/Au electrode samples are less pronounced in EW in comparison with the Teflon-coated ZnO NRs sample.

There are several factors that affect the change in WCAs, such as the droplet volume, droplet aspect ratio, dielectric thickness, dielectric constant, droplet conductivity, and roughness of the surface^42, 43^. In this study, we used DI water with a conductivity of 0.5 µS/cm, which is relatively high. For comparison, Zhou et al. used DI water with a conductivity of 0.05 µS/cm^44^. However, it is much lower than those of NaCl (537 µS/cm) and Na_2_SO_4_ (919 µS/cm) solutions^44^. Accordingly, it is reasonable to conclude that the conductivity of DI water used in this work was adequate to cause a relatively large change in WCA under the high voltage 250 V.

Via the application of a 250 V external voltage, the wetting of ZnO induces a change from a Cassie–Baxter state (non-wetting situation), in which air bubbles are entrapped inside the grooves underneath the water droplets, to a Wenzel state (wetting situation), in which water droplets fill up the roughness grooves completely. This behavior is largely due to the different surface energies above the Teflon-coated ZnO NRs and between the NRs, interacting with the water droplet under the application of a high voltage. Because the surface energy of the upper tips of the Teflon-coated ZnO NRs is lower than that of the interstitial spaces, the wetting state changes upon interaction of the water droplet with the different surface energies^23^.

According to Fig. 5a, WCA drops with increasing applied voltage almost linearly from 0 to 100 V, after which a much steeper linear relationship from 100 to 250 V is observed. The first stage of WCA drops, namely, from 0 to 100 V, is expected to be controlled by the structure and distribution of Teflon-coated ZnO NRs, while the second stage (100–250 V) is controlled by both the Teflon-coated ZnO NRs and the ZnO nano films. Based on Cassie’s model, the entrapped air bubbles between Teflon-coated ZnO NRs and ZnO nano films are the source of super hydrophobicity measured at zero applied voltage. Air is a very weak electrical insulator, and its breakdown voltage is about a few volts. Therefore, the leakage currents through these air bubbles almost instantly flow in all our experiments because of the much higher applied voltage. Subsequently, Cassie’s model breaks down, along with the source of additional hydrophobicity due to the formation of air bubbles. We believe this breakdown of Cassie’s model is a general feature of all EWOD systems. In fact, the Cassie–Baxter model calculates the effect of air pockets beneath the liquid droplet to a general wetting process, which was also used in many EW studies. But the Cassie–Baxter model falls short to explicitly account for the physics of loaded electrical fields to an EM wetting process. It is commonly accepted that air bubbles are trapped beneath the liquid droplets during the EW measurement, for instance Chen et al.^20^, reported that the water droplet/ZnO interface underwent transitions from the Cassie–Baxter to the Wenzel state in their EW experiments. However, the trapped air is a very weak electrical insulator and its breakdown voltage is only about a few volts. Direct EW can ionize the trapped air at low voltages before any observable changes in the WCA^1^. Therefore, contributions of the trapped air pockets to the change of wetting angle may be eligible. In summary, the Cassie–Boxer model is valid in EW studies but it falls short to reveal the influence of electrical field loads used in EW experiments. New models, like the one in this work, are called to introduce the physical drivers of electrical fields that facilitate the control of EWOD.

Optical images of water droplets, under different external voltages, on Teflon-coated ZnO NRs with diameters of 85 nm are shown in Fig. 5b. Two factors govern the droplet operation. The first is the driving force, which depends on the contact angle change under electrical voltage. The second is the resistance force of the surface on which the droplet moves^21^. Accordingly, when the applied voltage overcomes the resistance force, the droplet can change its shape to have a high contact angle. Because of the non-uniformity of the surface, nanostructure capillary and pinning effects, the WCA change should be limited under the applied voltage. However, in our case, Teflon was coated on the ZnO NRs. Therefore, the water droplet sat on the Teflon-coated ZnO NRs film and was not strongly anchored by the principles behind Cassie’s model. Because of this phenomenon, the change in WCA under quite a high applied voltage of 250 V could be facilitated.

For reverse bias, the applied voltages were 250, 200, 150, 100, and 50 V, resulting in WCA values of 76°, 90°, 99°, 120°, and 123°, respectively, as shown in Fig. 5c. A negligible change in WCA values between forward bias and reverse bias demonstrates the good EWOD performance of Teflon-coated ZnO NRs. Figure 5d shows the results of WCA hysteresis study. It can be seen that the WCA did not significantly change between the forward bias and reverse bias, demonstrating the good performance of the designed Teflon-coated ZnO NRs for EWOD applications.

We further discuss the influence of leakage currents on WCA using our recently developed NR-on-film wetting model^12^. Although we did not measure the exact leakage currents, these currents are expected for general metal oxides, as shown by the I–V loops in soda-lime glass^45^ or in SiO_2_^12^. As shown in Fig. 5a, there are two linear functions: one for 0 to 100 V, and the other for 100 to 250 V. We can explain these two linear behaviors as follows: (i) there are two types of nanostructures in the substrate: the ZnO thin films (100 nm-thick) and ZnO NRs (20 nm in dimension); (ii) each of these nanostructures has a unique current leaking initiation voltage; and (iii) the ZnO NRs are expected to be ionized at a lower initiation voltage compared to that for the ZnO film because of quantum confinement effects^46, 47^. Electrons in the NRs occupy higher energy levels than those of the excited electrons in the ZnO film because of their relative sizes (20 nm for NRs and 100 nm for thin films). Therefore, the first linear function in Fig. 5a is associated with leakage currents resulting from the Teflon-coated ZnO NRs, and the second function is from the ZnO films. Because the effects of leakage currents and substrate nanostructures are not included in the Lippmann–Young equation^12^, the current research may contribute to new design principles for advanced EWOD technology via intelligent control of substrate nanostructures.

The rate of WCA reduction changes at about 100 V is interesting, which may be explained by the two-stage activation of the composite nano ZnO structures. This proposed new concept (dependence between the rate of WCA reduction and the substrate nanostructure) may be applied to form new structure–design principles in EWOD research. We believe more sophisticated dielectric structure optimizations are necessary to bring down technical hurdles in developing industrial applications of EWOD. In brief, ZnO as a semiconductor material has an electron energy band gap of about 3.37 eV. Current flows through a ZnO semiconductor when the voltage applied across it exceeds the threshold voltage, which is much lower than the applied voltage of 250 V. In this experiment, there are two types of ZnO nanostructures, namely, ZnO nano film and ZnO NRs, which are in accordance with two classes of leakage currents. In turn, these leakage currents will contribute to a measured decrease in WCA values.

Although there are some studies on the EWOD properties of ZnO nanostructures, there are still some challenges, such as the relatively low breakdown voltages of the electrode/dielectric/water systems. In our previous paper^12^, using SiO_2_ nano-layers/Teflon system, we demonstrated: (i) reversible WCA, (ii) relatively high breakdown voltages, and (iii) a new NR-on-film wetting model to account for the observed changes in WCA. However, in this research, we explored new composite-ZnO NRs/Teflon structures, in order to achieve reversible WCAs and high breakdown voltages, guided by our new NR-on-film wetting model. In addition, we further validated our model by introducing the homo-composite dielectric structure, which is a composite of thin layered ZnO/Teflon and nano-roded ZnO/Teflon. In summary, our work advances the fundamental understanding of the EWOD principles and generates new practical techniques for reversible WCA changes in a large WCA range.

**Figure 7:**
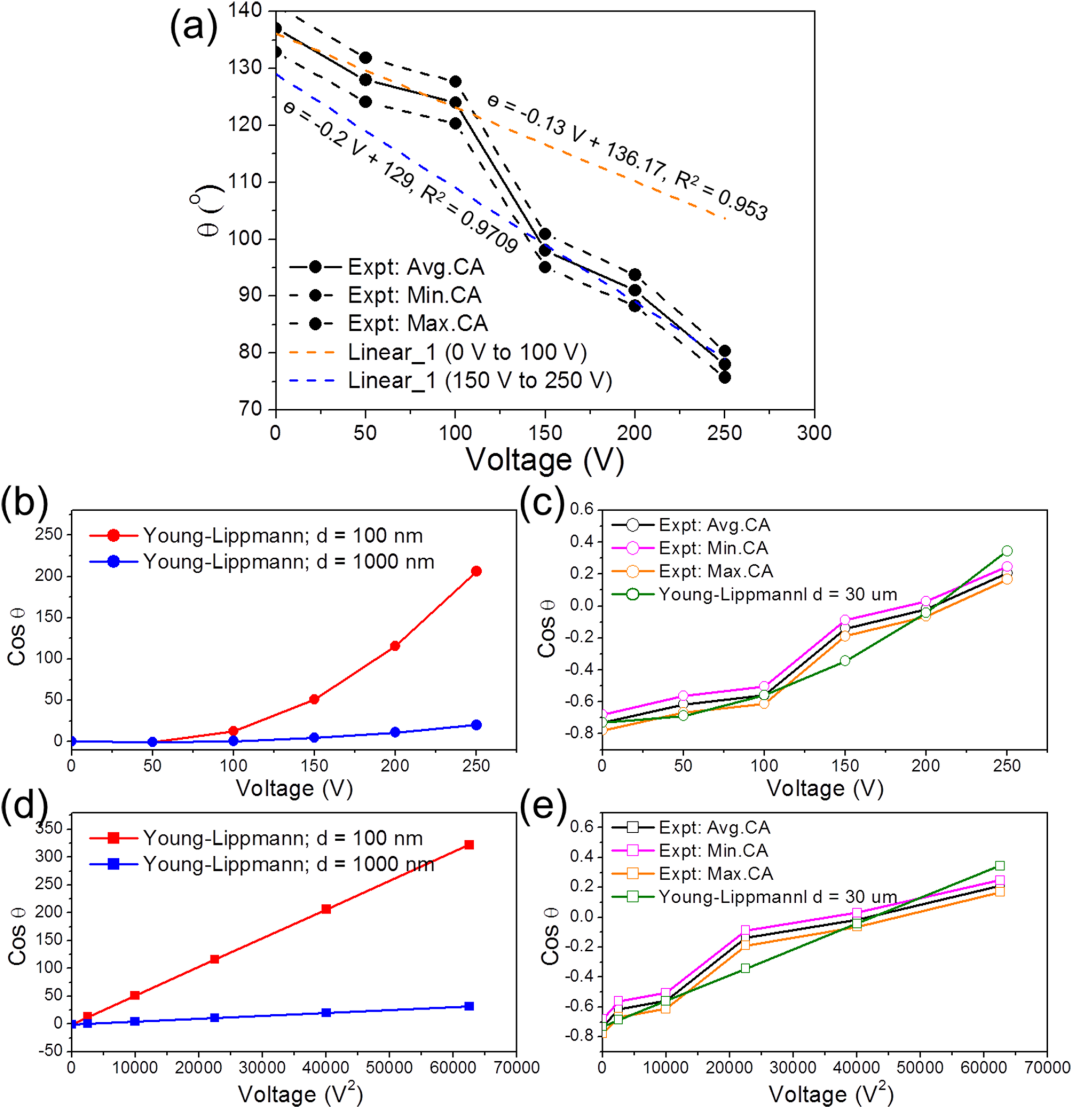
(**a**) WCA versus applied voltage. The points are from experiments. (**b**) The calculated numerical value of the “cos θ” term in the Young–Lippmann equation versus applied voltage (for two different “*d*” values). (**c**) The numerical value of the “cos θ” term from the best fitted Young–Lippmann equation (with d = 30 μm) versus applied voltage (V). (**d**) Plot of the numerical data of the “cosθ” term in the Young Lippmann equation to the square of applied voltage (V^2^) (for two different “*d*” values). (**e**) The best fitted Young–Lippmann equation (with *d* = 30 μm) versus square of the applied voltage used in the experiments.

To support the NR-on-film wetting model, quantitative analyses of the measured data are presented. Based on the Young–Lippmann equation, the only adjustable parameter in this study is the thickness of the film or the dielectric composite structure, as shown in Supplementary Table S3. Figure 7a shows the relationship between WCA and applied voltage. As shown, for voltages from 0 to 100 V, the linear equation between WCA and applied voltage (Eq. 2) is above the orange dotted line with R^2^ = 0.953

2 $$\theta \;(^\circ ) = - \;0.13 V + 136.17$$

For voltages from 150 to 250 V, the linear equation is above the blue dotted line with R^2^ = 0.9709.

3 $$\theta \; (^\circ ) = - \;0.2 V + 129$$

The slopes of the curves are not identical but very close since they represent the influence of applied voltages on the WCA on the same dielectrics (ZnO) but of different morphology [100 nm thick ZnO films for Eq. (2) and 100 nm thick ZnO film plus nanorod ZnO for Eq. (3)]. Therefore, these experiments are consistent with our proposed model which predicts a linear relationship between WCAs and the applied voltage.

Figure 7b shows plots of cosθ vs. applied voltage, by applying the Young–Lippmann equation at two different “*d*” values. Based on Supplementary Table S3 information and the Young–Lippmann equation, the only parameter that can be adjusted is the thickness of the dielectrics (*d*). By giving two values for “*d*”, namely, *d*_1_ = 100 nm and *d*_2_ = 1,000 nm, two distinctly different curves are calculated shown in red and blue, respectively. Since in our experiment, *d* = 100 nm (Fig. 2c), therefore, the values predicted by the Young–Lippmann equation do not represent the experimental data at all. In Fig. 7c, we compared the data obtained by the Young–Lippmann equation to our experiments. The best fitted equation was obtained when we took *d* = 30 μm, which is 300 times greater than the ZnO thickness observed in our experiments, indicating the calculated WCAs by the Young–Lippmann equation are not consistent with our experiments.

Similar to Fig. 7b, in Fig. 7d, we plotted the cosθ vs. V^2^ curve based on the Young–Lippmann equation. By taking *d*_1_ = 100 nm and *d*_2_ = 1,000 nm, two different curves were obtained as shown in red and blue lines, respectively. Since in our experiment, *d* = 100 nm, therefore, the values predicted by the Young–Lippmann equation do not represent the experimental data at all. In Fig. 7e, we compared the data obtained from the Young–Lippmann equation with our experiments data by plotting cosθ versus V^2^. The best fitted equation was obtained when we set *d* = 30 μm which is 300 times greater than the ZnO thickness observed in our experiments, again indicating the calculated WCAs by the Young–Lippmann equation are not consistent with our experiments.

Overall, it can be seen that the WCA vs. V relationship proposed by our NR-on-film wetting model satisfactorily represents our experimental data and further brings us new opportunities to characterize the nanostructures of dielectrics based on the changes of the slopes of WCA vs. V curves obtained in the EWOD experiments. Moreover, WCA calculations based on the traditional Young–Lippmann equation are not consistent with our experiments because the best-fitting model represents a dielectric thickness that is about 300 times higher than the value observed in our experiments.

## Conclusion

ZnO NRs with different diameters were prepared via the hydrothermal method and were subsequently coated with Teflon to increase the initial WCA. FE-SEM studies revealed the NR nature of the fabricated products. WCA measurements showed that the Teflon-coated ZnO NRs with diameters of 85 nm, prepared using the 0.1 M Zn^2+^ solution had the highest WCA, so these NRs were chosen for the EWOD studies. Different external voltages were applied to the fabricated EWOD platform, and it was found that the WCAs decreased with increasing voltage. In particular, for a voltage of 250 V, the wetting state of ZnO changes from a hydrophobic to a hydrophilic state, demonstrating large WCA change (59°), which is promising for the fabrication of EWOD devices. Furthermore, we proposed a new model to enable the design of advanced EWOD technology via nanostructure optimizations.

## Methods

To fabricate the EWOD platform, a thin layer of Au electrode was deposited on a glass substrate, and subsequently, a ZnO layer was deposited. ZnO NRs were then grown on these substrates and were finally coated by a thin Teflon layer. We also prepared the samples for comparison of EW behaviors: (a) Teflon-coated Au electrode (90 nm thick) and (b) Teflon-coated ZnO thin film (100 nm thick)/Au electrode (90 nm thick).

### Deposition of Au electrode

For EWOD studies, a metallic electrode is necessary, which is generally made from Au. Because the adhesion of Au to the glass substrate was not satisfactory, a 20 nm-thick Cr interlayer was initially deposited onto the glass via sputtering in order to increase the bonding force between the glass and Au. Pure Cr and Au targets with 2-inch diameter and 0.25-inch thickness were used for deposition using a sputtering system (Infovion Co. Korea). The sputtering conditions were as follows: target-to-substrate distance 10 cm, input power 100 W (for Cr) and 30 W (for Au), chamber pressure 10 mTorr, sputtering gas Ar, deposition temperature 25 °C, deposition time 30 s (for Cr) and 130 s (for Au). A 90 nm-thick Au thin film was then deposited on the glass substrate by the sputtering process.

### Deposition of ZnO layer

For depositing a thin layer of ZnO, atomic layer deposition (ALD) was employed. Water vapor and diethylzinc (Zn(C_2_H_5_)_2_, DEZn, DNF Co. Ltd., Korea) were used as precursors. ALD was performed at 150 °C and 0.3 torr. One cycle of ALD comprised 0.12 s pulsing for DEZn dosing, 3 s pulsing for N_2_ purging, 0.15 s pulsing for H_2_O dosing, and 3 s pulsing for N_2_ purging. ALD was continued until a 100 nm-thick ZnO thin film was obtained. The synthesized ZnO layer was then cleaned and dried thrice in alcohol and deionized water.

### Growth of ZnO NRs by hydrothermal method

ZnO NRs were successfully grown via the hydrothermal method. To obtain ZnO NRs with different diameters, zinc nitrate hexahydrate (Zn(NO_3_)_2_·6H_2_O, Sigma-Aldrich) solutions with different concentrations (0.03, 0.07, and 0.1 M) and 0.1 M hexamethylenetetramine ((CH_2_)_6_N_4_, HMT, Sigma-Aldrich) solution were stirred for 2 h and prepared for hydrothermal synthesis. The prepared solutions were mixed in a 1:1 volume ratio and then placed in a hydrothermal reaction vessel. The reaction time and temperature were 12 h and 150 °C, respectively.

### Teflon coating

A thin layer of a hydrophobic material such as Teflon is usually coated on the dielectric film to reduce the necessary external voltage for EWs and to increase the CA of the prepared samples^36^. A thin Teflon (SKB-TEFLON-100ML, Durasurf, Japan) film was coated by a spin-coater rotating at 4,000 rpm for 60 s. Thermal treatments at 80 °C were then carried out for 15 min to obtain a dense film. The thickness of the Teflon layer was about 20 nm.

### EWOD measurement

For WCA measurements during the EWOD studies, the substrates were put on a CA analyzer. Deionized (DI) water droplets (5 µL) were placed onto the ZnO NR surfaces. As shown in Supplementary Table S4^48–55^, EWOD devices actually can use any aqueous (polar) liquids, including most water-based biofluids and chemical solutions^56^. However, aqueous solutions, in general, are tested in EWOD studies, because they are often required for some EW applications, such as lab-on-chip (chemical properties) and liquid lenses (optical properties)^51^. In this study, we used DI water, which was produced from PURELAB UHQ (Siemens Water Technologies) apparatus and had an electrical conductivity of 0.5 µS/cm. However, because there are negligible charged ions in DI water, performance of EWOD with DI water is generally weaker than in the case with electrolyte solutions, where charge accumulation leads to a higher change in CAs^23^.

WCA measurements were performed using a custom-made measuring system. A Pt wire acted as the counter electrode, which was inserted into a droplet of DI water. Driving DC voltages (OPE-3002S, ODA Technology, Korea) in the range of 0–250 V were then applied between the tips of the Pt electrode (diameter 200 µm) and electrically grounded Au electrode. At each driving voltage, an image was captured using a camera (DFK 61AUC02, Imaging Source, Germany), and the WCAs were measured using IC Capture v2.1.109.576 software (Imaging Source, Germany). All experiments were performed in ambient atmosphere.

### Materials characterization

The morphology of the synthesized ZnO NRs was investigated via field-emission scanning electron microscopy (FE-SEM, Hitachi-S-4200) and transmission electron microscopy (TEM, TEM, Phillips CM-200). X-ray diffraction (XRD; Philips X’Pert diffractometer) with CuKα radiation (λ = 1.5418 Å) was used to study phase and crystallinity of a ZnO seed layer. Furthermore, atomic force microscopy (AFM, Bruker Nanoscope Multimode IV a) was used to obtain the surface roughness.

## Supplementary information

Supplementary Information.

## Data Availability

All the data are available from the corresponding author on reasonable request.
