# Supplementary material for: Electrowetting-on-dielectric characteristics of ZnO nanorods
Source: Sci Rep. 2020 Aug 25;10:14194. doi: 10.1038/s41598-020-71017-7 (PMC7447809; doi:10.1038/s41598-020-71017-7)
Supplement: Supplementary file 1 — Supplementary Information. [file 41598_2020_71017_MOESM1_ESM.docx]

**Supplementary Information**

**Electrowetting-on-Dielectric Characteristics of ZnO Nanorods**

Jae-Hun Kim^1^, Jae-Hyoung Lee^1^, Ali Mirzaei^2,3^, Hyoun Woo Kim^3,4,^*, Boon Teoh Tan^5^, Ping Wu^5,^* & Sang Sub Kim^1,^*

^1^Department of Materials Science and Engineering, Inha University, Incheon, 22212, Republic of Korea. ^2^Department of Materials Science and Engineering, Shiraz University of Technology, Shiraz, 71557-13876, Iran. ^3^The Research Institute of Industrial Science, Hanyang University, Seoul, 04763, Republic of Korea. ^4^Division of Materials Science and Engineering, Hanyang University, Seoul, 04763, Republic of Korea. ^5^Entropic Interface Group, Singapore University of Technology & Design, Singapore, 138682, Singapore. *email: sangsub@inha.ac.kr (S. S. Kim); hyounwoo@hanyang.ac.kr (H. W. Kim); wuping@sutd.edu.sg (P. Wu)

**Table S1.** Results of EWOD studies on different materials.

| Material | WCA ( ^o^) under no applied voltage (V) | WCA( ^o^) under Minimum applied voltage | WCA ( ^o^) under Maximum applied voltage | Ref. |
| --- | --- | --- | --- | --- |
| ZnO tetrapod | 130 | ~130 (10 V) | 85 (120 V) | S1 |
| ZnO thin film | 95.6 | 88.6 (35 V) | 79 (61 V) | S2 |
| ZnO NRs | ~160 | ~140 (2 V) | 90 (30 V) | S3 |
| Si_3_N_4_ film | ~110 | ~110 (30 V) | ~85 (180 V) | S4 |
| PFMA  (Poly-perfluoroctylmethacrylate) | 127 | ~125 (~4 V) | 85 (40 V) | S5 |
| La_2_O_3_@AAO  (Anodic aluminum oxide) | 100 | ~90 (60 V) | ~60 (76 V) | S6 |
| ZrO_2_/Teflon nanocomposite | 103 | ~100 (5 V) | 85.2 (60 V) | S7 |
| PDMS  (Poly-dimethylsiloxane)  microlens array | 140 | 120 (10 V) | ~60 (300 V) | S8 |
| AlN/Teflon film | ~110 | ~110 (10 V) | ~90 (40 V) | S9 |
| BST(Barium strontium titanate)/Teflon thin film | 114 | ~110 (10 V) | 72 (60 V) | S10 |

**References**

1. Xia, J. & Wu, J. Electrowetting on a dielectric surface roughened with zinc oxide tetrapod nanocrystals. *Physica E* **43**, 81–84 (2010).
2. Papadopoulou, E. L., Pahkozidis, A., Barberoglou, M., Fotakis, C. & Stratakis, E. Electrowetting properties of ZnO and TiO_2_ nanostructured thin films. *J. Phys. Chem. C* **114**, 10249–10253 (2010).
3. Wang, J. *et al*. Synergistic effects of the tip effect and electric adsorption on the enhanced electrowetting-on dielectric performance of structured ZnO surfaces. *Cryst. Eng. Comm.* **22**, 2361–2370 (2020).
4. Shen, H.-H., Chung, L.-Y. & Yao, D.-J. Improving the dielectric properties of an electrowetting-on-dielectric microfluidic device with a low-pressure chemical vapor deposited Si_3_N_4_ dielectric layer. *Biomicrofluidics* **9**, 022403 (2015).
5. Hou, J. *et al*. Electrowetting performances of novel fluorinated polymer dielectric layer based on poly(1H,1H,2H,2Hperfluoroctylmethacrylate) nanoemulsion. *Polymers* **9**, 217 (2017).
6. Jin, H. *et al*. The preparation of La_2_O_3_@AAO with simple hydrothermal method under ambient pressure and the enhanced electrowetting-on-dielectric performance. *Superlattice. Microst.* **110**, 233–242 (2017).
7. Hou, J. *et al*. Multiscale interface effect on homogeneous dielectric structure of ZrO_2_/Teflon nanocomposite for electrowetting application. *Polymers* **10**, 1119 (2018).
8. Im, M., Kim, D.-H., Lee, J.-H., Yoon, J.-B. & Choi, Y.-K. Electrowetting on a polymer microlens array. *Langmuir* **26**, 12443–12447 (2010).
9. Zhang, M. *et al*. Monolithic integrated system with an electrowetting-on-dielectric actuator and a film-bulk-acoustic-resonator sensor. *J. Micromech. Microeng.* **25**, 025002 (2015).
10. Sohail, S., Mistri, E. A., Khan, A., Banerjee, S. & Biswas, K. Fabrication and performance study of BST/Teflon nanocomposite thin film for low voltage electrowetting devices. *Sens. Actuators A* **238**, 122–132 (2016).

**Table S2.** Carrier concentrations of ZnO reported in the literature.

| Morphology | Growth Method | Carrier Con. (/cm^3^) | Ref. |
| --- | --- | --- | --- |
| Bulk ZnO | Hydrothermal (As-grown) | 2×10 ^10^-10^14^ | S11 |
| Bulk ZnO | Hydrothermal (Annealed at 930ºC) | 8×10 ^13^-2×10^15^ |  |
| Bulk ZnO | Hydrothermal (Annealed at 550ºC) | 2×10 ^13^-10^14^ |  |
| ZnO NRs (diameter=142 nm) | MOCVD | 4.95×10^17^ | S12 |
| ZnO NRs (diameter=96 nm) |  | 3.11×10^17^ |  |
| ZnO NRs (diameter= 85 nm) |  | 7.06×10^17^ |  |
| Pristine ZnO NRs | Hydrothermal | 7.86×10^16^ | S13 |
| Vertically well-aligned and epitaxial ZnO nanorod (diameter= 260 nm) | MOCVD | ~7.5×10^17^ | S14 |
| ZnO NRs (~ 150 nm) | Hydrothermal | 3.773×10^11^ | S15 |

Note: MOCVD: metalorganic chemical vapor deposition.

**References**

1. Kassier, G. H., Hayes, M., Auret, F. D., Mamor, M. & Bouziane, K. Electrical and structural characterization of as-grown and annealed hydrothermal bulk ZnO. *J. Appl. Phys.* **102**, 014903 (2007).
2. Yun, Y. S., Park, J. Y., Oh, H., Kim, J. J. & Kim, S. S. Electrical transport properties of size-tuned ZnO nanorods. *J. Mater. Res.* **21**, 132-136 (2006).
3. Rana, A. U. H. S. & Kim, H. S. NH_4_OH treatment for an optimum morphological trade-off to hydrothermal Ga-doped n-ZnO/p-Si heterostructure characteristics. *Materials* **11**, 37 (2018).
4. Park, J. Y. *et al*. Synthesis, electrical and photoresponse properties of vertically well-aligned and epitaxial ZnO nanorods on GaN-buffered sapphire substrates. *Appl. Phys. Lett.* **87**, 123108 (2005).
5. Chakraborty, M., Mahapatra, P. & Thangavel, R. Structural, optical and electrochemical properties of Al and Cu co-doped ZnO nanorods synthesized by a hydrothermal method. *Thin Solid Films* **612**, 49-54 (2016).

| Parameter | Symbol | Value | | |
| --- | --- | --- | --- | --- |
| Thickness of dielectric (variable) | d | 100 nm | 1 μm | 30 μm |
| Dielectric permittivity of vacuum (F/m) | ɛ_0_ | 8.85×10^-12^ | 8.85×10^-12^ | 8.85×10^-12^ |
| Relative dielectric permittivity | ɛ_r_ | 8.5 | 8.5 | 8.5 |
| Surface tension (N/m) | γ_LG_ | 7.28×10^-2^ | 7.28×10^-2^ | 7.28×10^-2^ |
| Specific capacitance (F/m^2^) | C | 7.52×10^-4^ | 7.52×10^-5^ | 2.51×10^-6^ |

**Table S3.** Parameters used in Young-Lippmann equation.

**Table S4.** Different droplets used for EWOD studies.

| Droplet used for EWOD study | Ref. |
| --- | --- |
| DI water | 49 |
| DI water | 50 |
| 1M KCL with DI water | 51 |
| 0.01 wt% [Sodium dodecyl sulfate](https://www.sciencedirect.com/topics/materials-science/sodium-dodecyl-sulfate) (SDS) with DI water | 52 |
| DI water | 53 |
| 3.0 M LiCl aqueous solution | 54 |
| Bi-distilled water | 55 |
| Distilled water | 56 |


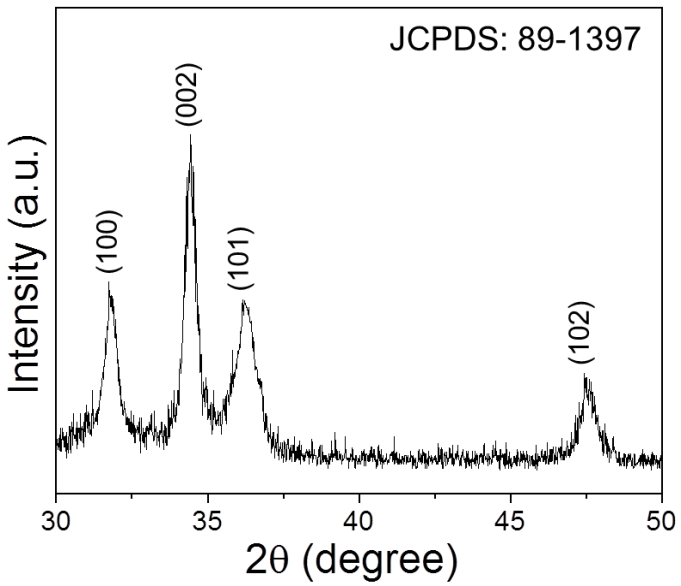


**Figure S1**. XRD pattern of ZnO NRs.

**
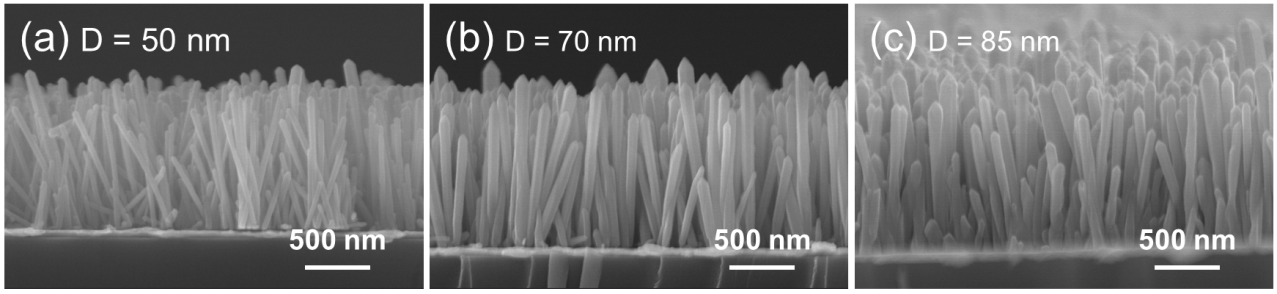
**

**Figure S2.** Cross-sectional FE-SEM images of ZnO NRs with different diameters of (a) 50 nm (initial Zn^2+^ concentration was 0.03 M), (b) 70 nm (initial Zn^2+^ concentration was 0.05 M) and (c) 85 nm (initial Zn^2+^ concentration was 0.1 M).


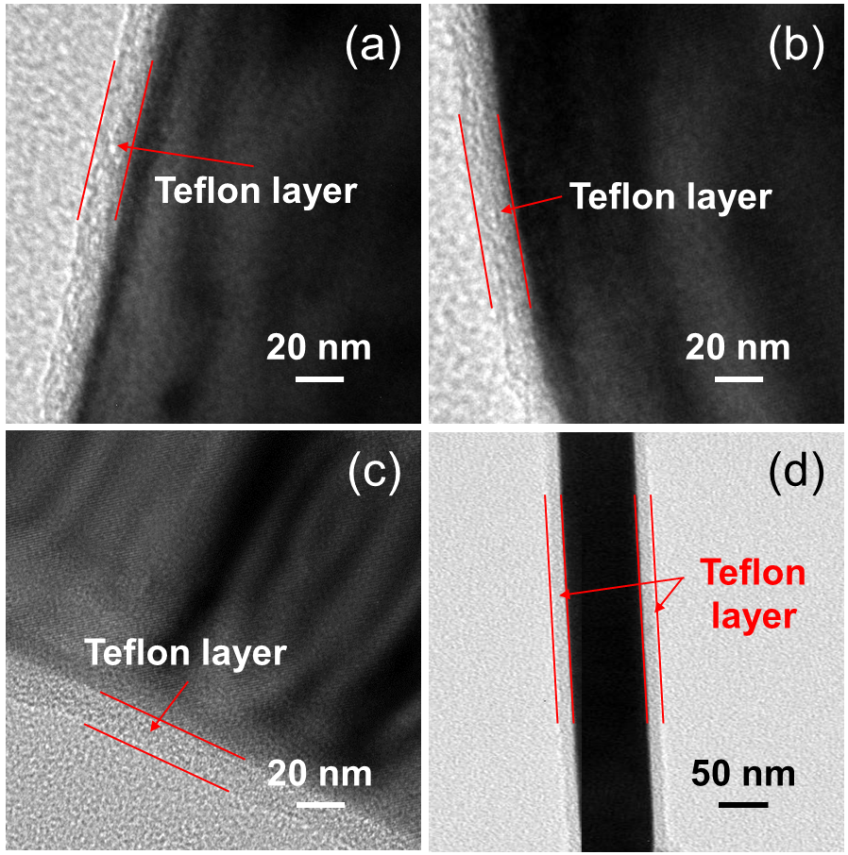


**Figure S3**. (a)-(c) TEM images with same magnification, showing coating of a uniform Teflon layer on the surface of randomly selected ZnO NRs. (d) lower magnification TEM image showing deposition of the uniform Teflon layer on the surface of a randomly selected ZnO NR.


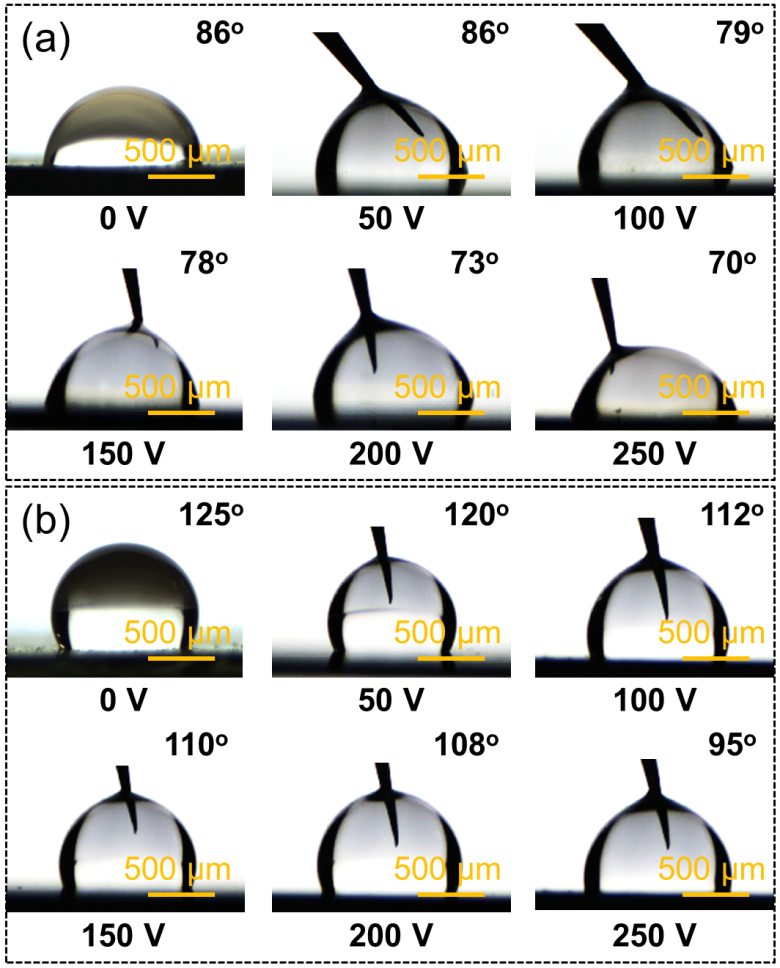


**Figure S4.** Optical images of water droplets on ZnO NRs with diameters of (a) 50 nm and (b) 70 nm, under different external voltages. Magnification 5X.
